# Annotations

**Published:** 1889-07-13

**Authors:** 


### Annotations.

**"Voiceless Wives."** "Happy the cicadas live, since they all have voiceless wives," says Xenarchus. The cicada is a well-know tropical insect, the females of which are mute, though the males produce a sound which may be heard a mile off. It is said that the Greeks formerly kept, and that the Chinese still keep, these insects in cages for the sake of their song, which, however, is not a song like that of the birds, but a "stridulation" produced by the vibration of a membrane set in action by a special muscle. But however noisy the males may be the females are mute, and Xenarchus congratulates the cicada gentlemen on that account. Whatever else civilisation may be doing for mankind, it seems to be improving the shrewish woman off the face of the earth. The female scold in ancient times was so common that she passed into a by-word. Socrates, as everybody knows, had to submit to be lectured by Xantippe; Xenarchus, in all probability, had a wife still worse. Herodias' daughter, who so promptly asked for the "head of John the Baptist on a charger," must have been one of those robustly-nerved young women whom nothing could reduce to the level of a swoon. Milton seems to have had but an indifferent time of it with his women-kind, and John Wesley formed a very poor opinion of the sex after his experience of married life. The old view of women—that they were made for the convenience and pleasure of men--did not by any means "work" well. The more recent and modern estimation of them is not only far more scientifically just, but it "works" much better. Since women began to be educated as intelligent beings, the shrew and the scold have to a large extent died out from among the cultured classes. Even the mother-in-law has learnt to put away suspicion and jealousy, and to treat her daughter's husband with benevolent consideration. Among the poor it is to be feared that rancorous tongues still drive husbands to public-houses, and sons and daughters to seek their pleasure away from home. It is evident, therefore, that the better education which modern women receive has had the delightful effect of softening asperities of temper, developing a sweet reasonableness, and making the sex as much more delightful than their grandmothers, as they are more educated and companionable. This is a practical test and proof of the fitness of women for education and of its value to them. Few modern men who are married to cultivated women endorse the view of Xenarchus, "Happy the cicadas live, since they all have voiceless wives."

**Convocation and Gambling.** Last week the members of the Lower House of Convocation devoted themselves seriously to the consideration of the prevalent vice of gambling. It was generally admitted that, among the middle and lower classes especially, betting and gambling are increasing to a very marked extent. No physiologist would deny that the excitement and anxiety which accompany betting and gambling are seriously injurious to health. The average bookmaker on the turf suffers from physiological aberrations almost peculiar to himself. The excitable brain, the palpitating heart, the sleeplessness, which generally spring from over-mental strain, are often very pronounced in him. There is hardly a diocese in England, it is said, except Truro, in which gambling is not more widespread than at any previous time. The mining counties are notorious for the number and variety of their gamblers and their betting games. This is a serious danger to national health as well as to national character. Convocation passed a resolution urging the clergy to protest both privately and from the pulpit, and in "every other way within their power to discourage the present practice." It was said that the upper classes have not shown the same downward tendency as the middle and the lower. We should take leave to doubt that. But whether or no, it is precisely in such things as betting and gambling that the lower classes can most easily associate with and imitate their social superiors; and the responsibility of the latter is therefore correspondingly increased. It is eminently an upper class question. If that class could be prevailed upon to give it up and to discountenance it as a vulgar, greedy, and shameful form of amusement, there is no doubt that a complete reformation might be looked for all over the country. Some warm speaking took place when it was proposed by the Rev. J. J. Jeffcock to add a rider, "Discouraging the employment of raffles and lotteries at bazaars and fancy fairs." Strange to say, there were some clergymen present who, whilst opposed to gambling in any other way, objected to this very sensible and much-needed rider. Among those was the Bishop of Colchester. The House, however, we are glad to note, carried the rider as well as the resolution. How the Bishop of Colchester, or any other clergyman, can fail to see that the raffles and lotteries of bazaars and fancy fairs belong absolutely to the same category as any other kind of gambling passes comprehension. His lordship and others seemed to think that the end somehow sanctified the means—a most dangerous and unchristian idea. Many a bookmaker follows his occupation for no other purpose than that of earning a living for his wife and family. Is not that a legitimate and virtuous end? But will the bishop contend that it justifies the means? All honest and patriotic citizens should unite in an earnest effort to persuade or compel the people to abandon gambling as a practice which is as dangerous to health and prosperity as it is undoubtedly vicious in itself.

**Pure Air for Dyspeptics.** When a person is given to dyspepsia, as many people are by inherited constitution, and many more from acquired habit, a great variety of circumstances may lead to recurrent attacks. With some people an impure atmosphere, or hot, sunny, and exhausting weather, or extremely cold, chilly weather, will be quite sufficient to encourage the approach of the enemy and to throw wide the doors for him to enter. If a confirmed dyspeptic falls into a lower than ordinary state of general health, his one persistent foe has it practically all his own way and makes life a perfect misery. Some dyspeptics have found out by experience that a change of air from town to country or sea, or from a hot, relaxing climate to a colder one will drive away their distressing symptoms in a few days. Bad air, or air that though not bad for the robust is very far from purity, cannot make the blood pure like good air; it cannot get rid of the waste tissues of the body so completely as they should be got rid of; it does not stimulate and brace up the spirits; it does not promote sleep. On the other hand, in order that digestion may be comfortable and perfect, there must be a sufficient quantity of effective gastric juice secreted after every meal. But this requires pure blood, and a nervous system in sound, working order. By far the best thing for many dyspeptics to do is to seek an immediate change of air. It is often said of drugs, or rather it used to be often said of them, that they "acted like charms." Very few drugs indeed have anything of the "charm" about them. But a pure, clear, bracing atmosphere makes such a change in the blood and nervous system in a few hours that it almost deserves to be spoken of as a "charm." There are many people in London every summer who make up their minds to stay at home instead of having their usual two or three weeks in the country or at the sea. They think they cannot afford the time, or they cannot spare the money, or they feel well enough to do without going away. It is a grave mistake. Nothing but absolute necessity should keep the hard-working father, or the mother of many cares, or the children who have been cooped up in school, from the pure and life-giving breezes of the mountain or the sea. It is better to dress more plainly, or to drink less wine, or to do without dinner-parties in the winter than to make an unbroken stay in London for a whole twelve months. Health, efficiency, and pleasure, at any rate for the dyspeptic, often turn on the question of pure air, and plenty of it.

